# Development of Liquid Diene Rubber Based Highly Deformable Interactive Fiber-Elastomer Composites

**DOI:** 10.3390/ma15010390

**Published:** 2022-01-05

**Authors:** Vikram G. Kamble, Johannes Mersch, Muhammad Tahir, Klaus Werner Stöckelhuber, Amit Das, Sven Wießner

**Affiliations:** 1 Research Division Elastomers, Leibniz-Institut für Polymerforschung Dresden e.V. (IPF), 01069 Dresden, Germany; tahir@ipfdd.de (M.T.); stoeckelhuber@ipfdd.de (K.W.S.); das@ipfdd.de (A.D.); wiessner@ipfdd.de (S.W.); 2 Institute of Materials Science (IfWW), Faculty of Mechanical Science and Engineering, Technische Universität Dresden, 01062 Dresden, Germany; 3 Institute of Solid State Electronics, Technische Universität Dresden, 01062 Dresden, Germany; johannes.mersch@tu-dresden.de; 4 Institute of Textile Machinery and High Performance Material Technology, Technische Universität Dresden, 01062 Dresden, Germany

**Keywords:** fiber-rubber composites, SMAs, soft actuators, smart composite structures

## Abstract

The preparation of intelligent structures for multiple smart applications such as soft-robotics, artificial limbs, etc., is a rapidly evolving research topic. In the present work, the preparation of a functional fabric, and its integration into a soft elastomeric matrix to develop an adaptive fiber-elastomer composite structure, is presented. Functional fabric, with the implementation of the shape memory effect, was combined with liquid polybutadiene rubber by means of a low-temperature vulcanization process. A detailed investigation on the crosslinking behavior of liquid polybutadiene rubber was performed to develop a rubber formulation that is capable of crosslinking liquid rubber at 75 °C, a temperature that is much lower than the phase transformation temperature of SMA wires (90–110 °C). By utilizing the unique low-temperature crosslinking protocol for liquid polybutadiene rubber, soft intelligent structures containing functional fabric were developed. The adaptive structures were successfully activated by Joule heating. The deformation behavior of the smart structures was experimentally demonstrated by reaching a 120 mm bending distance at an activation voltage of 8 V without an additional load, whereas 90 mm, 70 mm, 65 mm, 57 mm bending distances were achieved with attached weights of 5 g, 10 g, 20 g, 30 g, respectively.

## 1. Introduction

In the field of soft robotics, a variety of elastomer-based smart materials have become very interesting candidates to achieve large recoverable deformations and for combined use with inherently implemented specific core functions such as sensing, actuation, or adaptive stiffness/damping control [1,2,3,4,5]. The combination of elastomeric matrices with tailored reinforcing and functional textile structures has been demonstrated to be a capable technology in terms of introducing (anisotropic) reinforcement to the otherwise very compliant soft elastomeric structures. Some examples include the development of highly deformable mechanisms such as parabolic antenna [6], in addition to the production of switchable covers and stoppers, parts for influencing fluid flow [7], or shape memory alloy (SMA)-actuated planar sandwich structures, such as those developed in [8,9]. Such elastomer-fiber composites have become a promising approach to the generation of controllably deformable components with specifically adjustable properties. The mentioned examples are almost exclusively based on an elastomer matrix that contains an additional cured Polydimethylsiloxan-Elastomer (PDMS, silicone rubber) with a low precursor-viscosity in order to achieve a good infiltration when manufacturing the sandwich-structures by infusion or gravity-driven pouring technologies [10,11]. In addition to the widely investigated PDMS, which can also serve as dielectric elastomer actuators (DEA), reactive polyurethanes (PU) and polyacrylates are also reported to be alternative elastomer matrices.

On the other hand, in recent years, the capability of printable functional liquid silicone elastomer precursors for the additive manufacturing of 3D and 4D structures has been widely reported in the scientific literature [12,13], thus enabling the preparation of adaptive grippers, highly deformable vents, etc., with embedded actuation and strain-sensing functions that rely on magnetostrictive stiffness adaption and piezoresistive sensing principles [14,15,16]. Commonly, these functionalities are incorporated into the PDMS elastomers by adding functional micro- or nanosized fillers such as magnetizable (hard or, soft magnetic, ferromagnetic) particles (Carbonyl iron particles (CIP), Magnetites, etc.) or electrically conductive fillers, i.e., carbon allotropes (highly structured carbon blacks, Graphene Nanoplatelets (GNP) and Carbon Nanotubes (CNT)), silver nanowires, etc. In the scientific and patent literature, there are also limited reports on the utilization of liquid diene elastomers in additive manufacturing technologies. Strohmeier et al. [17] reported on the additive manufacturing of modified photosensitive Liquid Isoprene Rubber, where digital light processing was used for curing to create complex geometries. Due to the UV-induced curing reaction, the use of carbon black as functional or reinforcing filler is largely restricted because of the broad light absorption spectra in this process. In a patent, Yoshinaga et al. [18] presented different structures and lattices that were additively manufactured from formulations of liquid isoprene rubber radically cross-linked with urethane acrylate oligomers that can contain up to 30 phr of silica filler. Furthermore, only very recently, Thiel et al. [19] described the design of a novel additive manufacturing printing head for the processing of liquid rubber polymer.

The very small number of recent reports on the development of functional composites based on solid rubbers focus on oil-extended ultra-soft natural rubber (NR)-based strain sensors for soft robotics [20], the electro-polymerization of liquid rubber based pressure sensor assemblies [21], and our recent work demonstrating the possibility of combining the magnetostrictive functionality of the elastomer matrix with controllable large deformability through the successful preparation and testing of textile–elastomer-sandwich structures composed of magnetostrictive solid nitrile butadiene rubber (NBR) and functional flat-knitted reinforcing glass fiber fabrics, with integrated SMA wire fixed using the tailored fiber-placement technology [5]. However, the latter approach is limited to simple 2D-geometries, specifically only flat/planar structures, since the consolidation of the sandwich structure and the crosslinking of the elastomer components has to be carried out in a vulcanization press at high pressures of >30 bars. In combination with the high viscosity of the uncured rubber (in the order of several 10,000 Pas), this imposes massive restrictions for the manufacturing of composites of more complex shapes or the implementation of complex functional 3D textile geometries. Compared to solid rubbers, liquid rubbers have the capability to impregnate and inter-lock the reinforcing and functional textile structures in order to attain improved adhesion in complex textile-based smart systems.

In this research work, we intended to develop an interactive fiber-elastomer composite, the matrix of which is entirely based on liquid diene elastomers. In contrast to silicone elastomers, cross-linked diene rubbers exhibit advantages in terms of their mechanical properties, particularly a higher resistance against crack initiation and crack propagation under cyclic and dynamic deformation [22]. Similarly to solid diene rubbers, the liquid counterparts are crosslinkable with peroxide or sulfur curing systems for the tailoring of load-bearing characteristics by means of carbon–carbon or sulfidic linkages between chain segments. Considering the future prospects of liquid diene rubbers, functional elastomeric derivatives with upgraded chemical and physical properties can be prepared through chemical modifications involving carbon–carbon double bonds in this largely unexplored class of rubbers. Liquid diene rubbers thus represent a promising prospect for use in soft intelligent structures exhibiting large deformations, contrary to earlier reports on stiff fiber-reinforced-plastic-based adaptive structures with very limited deformability [23]. For the present work, a liquid polybutadiene rubber (LBR) was investigated in relation to the development of a smart planar configuration of an interactive textile-rubber composite structure. Herein, the motion tasks were performed by a shape memory alloy (SMA) wire integrated onto the reinforcing textile component using the tailored fiber-placement technology. The shape memory alloy wires actuated upon conversion of thermal energy into mechanical energy and demonstrated contraction movements with high actuation force upon transformation from a martensite to an austenite crystalline structure. Such multi-material systems can be integrated into soft elastomeric matrices with signal processing units to achieve textile-reinforced adaptive or smart structures. These smart structures can create adjustable large movements through control systems using feedback signals. As per our knowledge, no previous study has reported on the use of liquid polybutadiene rubber as a matrix elastomer for the development of highly deformable interactive fiber-elastomer composite-based soft intelligent structures that exhibit bending movement, which was the goal of this work.

## 2. Materials and Methods

### 2.1. Materials

A liquid polybutadiene-based rubber was selected for the investigations (see Table 1). The chosen liquid diene rubber is a high-molecular-weight and low-vinyl (<5% content) butadiene of homopolymer grade, and is commercially available as LBR-300 from Kuraray Europe GmbH, Hattersheim, Germany. For crosslinking of the LBR-300, a sulfur curing system was used, which included high-purity sulfur and zinc oxide from Acros Organics N.V. (Geel, Belgium), stearic acid from Fisher Scientific GmbH (Schwerte, Germany), and N-cyclohexyl-2-benzothiazole sulfenamide (CBS), diphenylguanidine (DPG), and ultra-fast accelerator zinc dialkyl dithiophosphate (Rhenocure ZDT/S) from Rhein Chemie GmbH (Mannheim, Germany). In a preliminary study, the amount of sulfur was varied from 1.8 to 2.4 phr and, subsequently, the curing behavior was studied by vulcametry, and the tensile properties of the vulcanizates were also examined. An amount of 2.2 phr showed the best balance in terms of achieving fast curing, the required crosslinking density, and a suitable stiffness–deformability ratio of the vulcanizates. The formulations of the used rubber mixture are shown in Table 2.

A functionalized fabric was used as a reinforcement textile and as a stimuli-responsive shape-changing intelligent structure to prepare the soft adaptive planar structure: a shape memory alloy (SMA) wire of 0.305-mm thinness was embroidered onto a classical biaxial woven glass-fiber fabric (Lange + Ritter GmbH, Gerlingen, Germany) to obtain a functional textile specimen. In line with the information reported in the product data sheet from Memry Corporation (Freiburg, Germany), the SMA wire (Nitinol) exhibited most of the martensite-to-austenite phase transformation over a narrow temperature range of 95 to 110 °C and, in so doing, manifested a substantial deformation force that contributed to the reversible shape memory effect.

**Figure 1:**
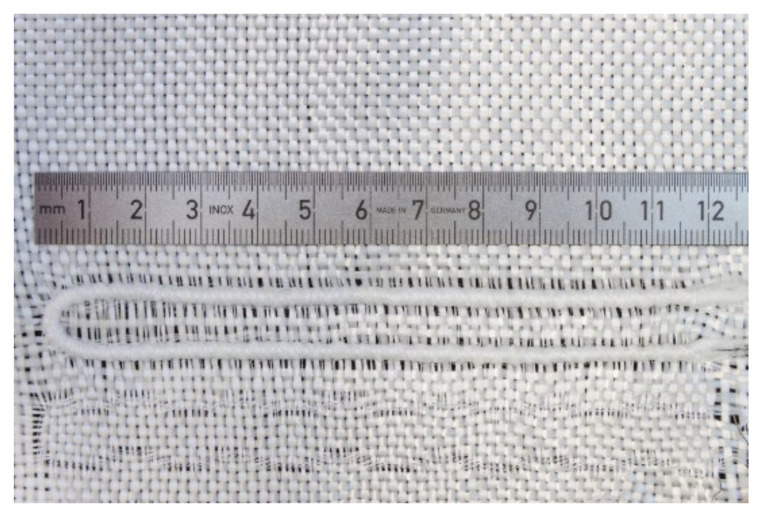
Polypropylene-glass fiber braided SMA wire loop integrated onto the glass fiber fabric.

Before embroidery, the SMA wire was braided with a combination of polypropylene and glass fiber yarns by means of a friction spinning process, as described in Ashir et al. [24]. Braiding created a core–shell structure wherein the inner core consisted of SMA wire, the middle shell was of glass fiber, and the outer fuzzy shell was composed of polypropylene, which ensured a mechanical interlocking with the liquid rubber matrix. The braided SMA wires were embroidered onto the woven fabric at a defined position by means of a tailored fiber-placement technique, without twisting and distortion of the interlacing angle of the warp and weft yarns of the fabric, as shown in Figure 1. Embroidering anchored the position of the active SMA wire in the smart structure to achieve a repeatable and controllable deforming motion.

### 2.2. Preparation of Planar Smart/Adaptive Structure

Before mixing with the liquid rubber, the solid components, such as sulfur and zinc oxide, as well as the accelerators, were manually pestled in a mortar for 5 min. Afterwards, all the ingredients of the formulation shown in Table 2 were placed together in a plastic jar and mixed using a SpeedMixer (DAC150 SP by Hauschild GmbH & Co. KG, Hamm, Germany) with the following mixing sequence, [rotational speed (time)]: 800 rpm (5 s), 2500 rpm (120 s), 1200 rpm (5 s), 2500 rpm (100 s), 800 rpm (5 s). This mixing sequence was repeated three times to produce a homogeneous mixture.

A moving die rheometer (Scarabaeus SIS V-50, Scarabaeus Mess- und Produktionstechnik GmbH, Wetzlar, Germany) was used to characterize the crosslinking behavior of the liquid polybutadiene rubber mixtures.

For characterization of the basic properties, the LBR mixture was compression molded to a sheet of 2-mm thickness using a metal frame and crosslinked to its respective optimal cure time T90, which was derived from the crosslinking studies. After storing at room temperature for 24 h, dumb-bell specimens S2, produced according to the DIN 53504 standard, and rectangular specimens of 35 mm × 10 mm, used for dynamic mechanical analysis, were punched out using specific dies. Tensile testing was conducted using a universal testing machine (Zwick 1456, Z010, ZwickRoell GmbH & Co. KG Ulm, Germany) at a crosshead speed of 200 mm/min according to the DIN 53504 standard. The testing machine was equipped with optical strain sensors and a 1 kN force transducer. Three specimens were tested and average values of tensile properties were derived, as well as stress–strain data, which were later used for parametrization of the Ogden model for the simulation.

To characterize the temperature-dependent dynamic mechanical behavior, temperature sweep tests were carried out on the rectangular specimens using a DMA mechanical spectrometer (GABO EPLEXOR 150N, Gabo Qualimeter GmbH, Ahlden, Germany) in tension mode at a pre-strain of 2% and a dynamic strain of 0.5%, 2 K/min heating rate (temperature range of −120 to 100 °C) and a frequency of 10 Hz.

**Figure 2:**
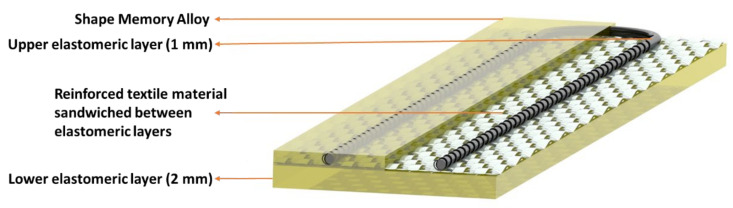
Graphical image showing the eccentric position of SMA-integrated textile in a multilayer actuator assembly.

The functional textile specimen (120 × 80 mm) of Figure 1 was eccentrically embedded in the rubber mixture for a stack of three layers using an assembly of rectangular metallic frames. This setup of a stack of layers was subjected to a low-temperature compression-molding process to crosslink the liquid rubber in a vulcanization press (TP 1000 by Fontijne B.V., Delft, The Netherlands). The crosslinking of rubber was realized at an unusually low temperature of 75 °C to avoid the phase transformation of shape memory alloy wire during the compression-molding and vulcanization process. The kinetics of the rubber crosslinking was much slower at 75 °C and the optimum cure time was around 3 h (described in more detail in the Results and Discussion section). The graphical representation of the molded assembly is shown in Figure 2. The thicknesses of the upper and bottom elastomeric layers are 1 mm and 2 mm, respectively.

**Figure 3:**
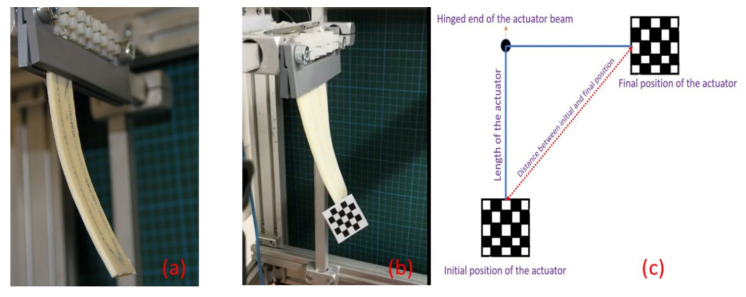
Actuation setup: (**a**) specimen of LBR-based interactive fiber-rubber composite clamped and with the SMA wires connected to the voltage supply via a screw terminal; (**b**) the bending of the structure was monitored by optical markers; and (**c**) the bending distance was defined as shown.

The cyclic deformation of the adaptive composite structure was characterized using the setup shown in Figure 3a: The specimen of the actuator assembly was clamped via a screw terminal which simultaneously provided the electrical contacting of the SMA-wire ends. The screw terminal was connected to a programmable power supply that made it possible to set controllable voltage cycles of specified durations. For the actuation experiments, the arbitrary power supply function was set to apply different voltage levels (i.e., 5, 6, 7 or 8 V) for 3 s and then to sink to 0 V for 10 s. The number of repetitions was set to 5 cycles in the current experiments. The actuation behavior, i.e., the voltage- and time-dependent bending of the structure, was monitored by optical markers, which were placed at the lower end of the specimen (Figure 3b), and as a measure to define the actuation, the distance was evaluated, as shown in Figure 3c.

## 3. Results and Discussion

The unsaturated liquid polybutadiene rubber is crosslinkable by the sulfur curing system where the kinetics of the crosslinking reaction can largely be controlled by specific chemical compounds known as accelerators. The presence of the SMA wire in a multi-component smart composite restricts the use of vulcanization temperatures higher than the SMA activation temperature of 95 °C; thus, the typically used vulcanization temperatures of 150–170 °C cannot be applied. Therefore, a rubber formulation needs to be developed to crosslink the liquid rubber by using sulfur curatives at a low temperature of 75 °C only. The conventional guanidine and sulfenamide accelerators alone are inactive at such low temperatures. However, in combination with a super-fast thiophosphate accelerator, diene rubbers can be crosslinked even at such low temperatures as 75 °C, as is the case in the present study. The combination and ratio of the accelerators and the sulfur determines the curing behavior (scorch time and cure time), network structure, and material properties, especially for the low-temperature thiophosphate-cured formulation.

**Figure 4:**
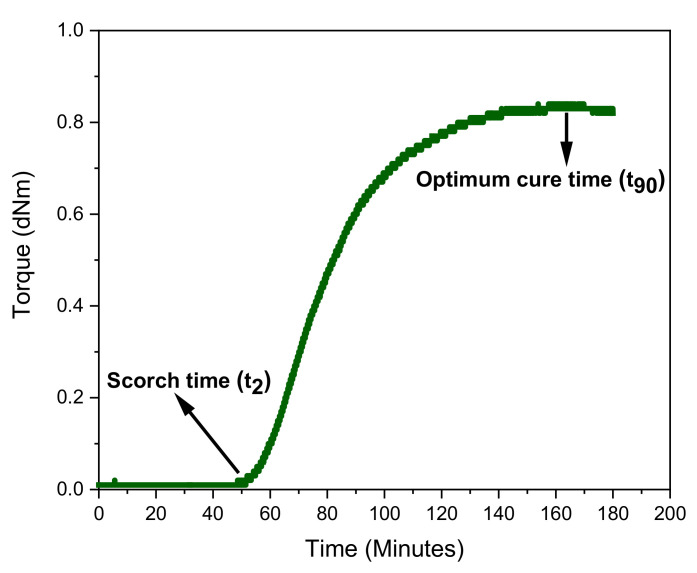
Vulcanization isotherm of the LBR-matrix at 75° with indications of scorch time and optimal cure time.

Table 2 shows the rubber formulation used for crosslinking the LBR matrix of the soft planar structure at 75 °C. The curing curve of this rubber mixture measured in the moving die rheometer reflects that the crosslinking process started and proceeded very slowly to reach an equilibrium torque value, which was related to a constant and stable network chain density towards the end of rheometer test at 75 °C (see Figure 4). The kinetics of the crosslinking reaction was slow as it depended, in addition to the curing chemicals, on the temperature of vulcanization. The scorch and cure times were around 55 and 170 min, respectively, for the selected delayed-action accelerator system. The longer scorch safety time was essential to avoid premature vulcanization of the rubber matrix in order to produce complex multi-material sandwich constructions via the subsequent stagging of functional layers. Before the onset of curing, the LBR mixture exhibited a very low viscosity, as indicated by the very low level of the torque in the first 50 min of the curing test. This indicates that the desired easy flowability of the formulation, that was required in order to impregnate the fabric, was achieved, and thus, the results obtained for this mixture were in clear contrast to those associated with typical solid rubber-based compounds. Thus, it can be stated that the availability of an easily flowing and low-temperature curing recipe is a pre-requisite of utmost importance for the production of the elastomer matrix of a soft composite structure with integrated SMA actuation, and that this was successfully achieved, as evidenced by the development of the torque in the isothermal rheometer test.

**Figure 5:**
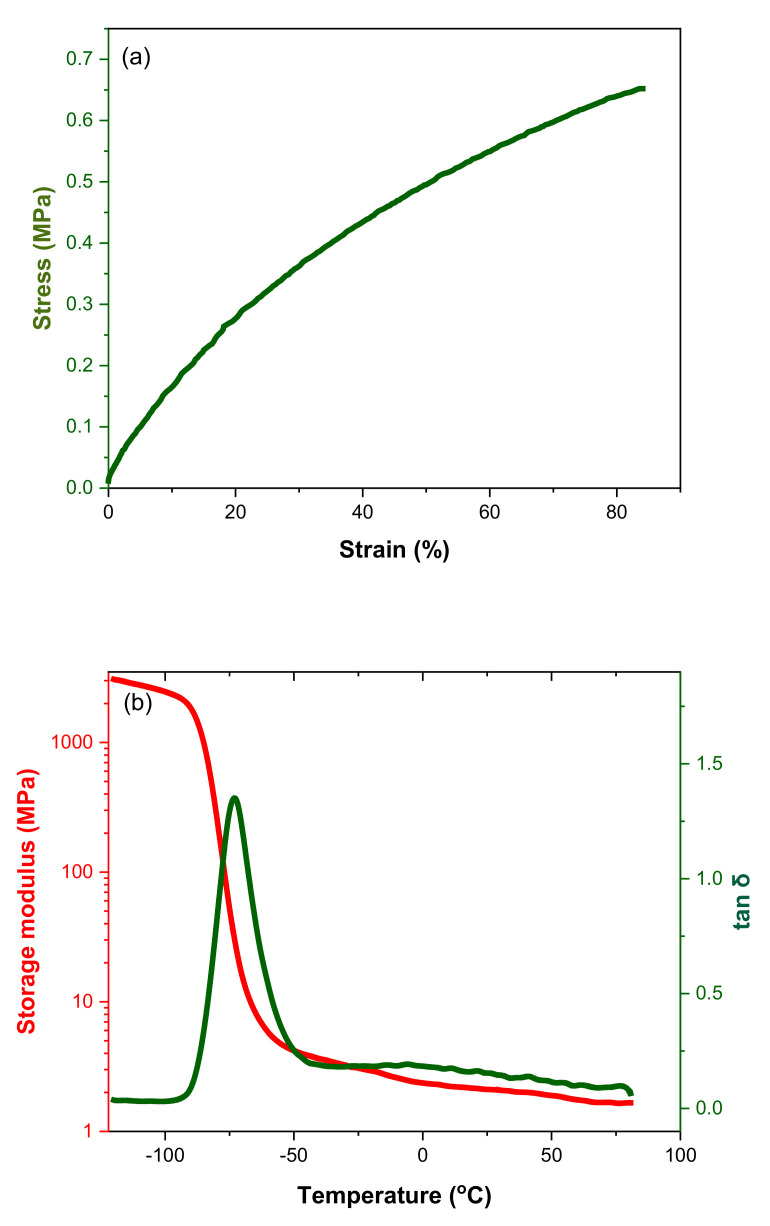
(**a**) Stress-strain behavior of the LBR cured at 75°. (**b**) Storage modulus and mechanical loss factor.

The stress–strain curve of the cured LBR formulation obtained under uniaxial tensile loadings is shown in Figure 5a. The soft and otherwise non-reinforced polybutadiene rubber demonstrated a nonlinear elastic response and deformed by about 20% upon the application of only 0.25 MPa of tensile load. This shows that the LBR elastomer could be deformed by very low forces, and thus, it almost did not restrict the bending deformation of the SMA-functionalized textile in order to attain large deformations in the smart structure. However, this also implies that the capability to store elastic deformation energy was rather low, and thus, also did not support the re-deformation of the composite structure after actuation.

A simple temperature-sweep dynamic mechanical test was performed to characterize the structural relaxation processes in addition to the segmental relaxations that mark the glass transition temperature of the rubber. Figure 5b shows that the glass transition temperature of the crosslinked polybutadiene rubber was around −65 °C, indicating the applicability of using such soft rubber actuators in sub-zero conditions. The storage modulus values vary with the applied temperature. In contrast to solid rubber vulcanizates, the storage modulus was at a considerably low level of ~2 MPa and reflected the very loose crosslinking of the LBR. Thus, the LBR-elastomer matrix provided the pre-requisites for allowing largely unconstrained deformation of the interactive fiber-rubber composite. The tan δ values above Tg confirmed that the predominant behavior of the LBR-vulcanizate was that of a viscoelastic solid viscoelastic with only low amounts of dissipated energy. However, the small local increase in tan δ at about 0 °C could be an indication of relaxation processes related to disentanglements of the polybutadiene molecules (and thus, a deviation from the typical hyperplastic network-like behavior), which can, most likely, be attributed to the low network density achieved in this LBR with the presence of a high amount of dangling chain ends. Such influence on dissipative loss was previously reported for a functionalized liquid butadiene rubber-modified SSBR tire formulation [25] However, this did not negatively influence the deformation behavior of the composite structure, as shown in the following section.

**Figure 6:**
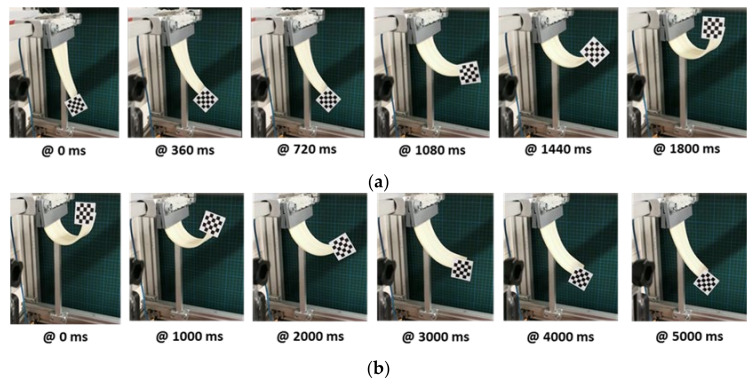
Actuated deformation (bending) of the interactive fiber-elastomer composite with respect to time. (**a**) Voltage-actuated bending and (**b**) re-bending after actuation.

The actuated deformation and re-deformation behavior of the adaptive composite are shown in Figure 6a,b, respectively.

Upon the application of voltage, the passage of electrical current through the SMA wire resulted in joule heating and, thus, initiated the transformation from the austenite to the martensite crystalline phase. This contracted the SMA wire and subsequently bent the adaptive planar composite structure.

**Figure 7:**
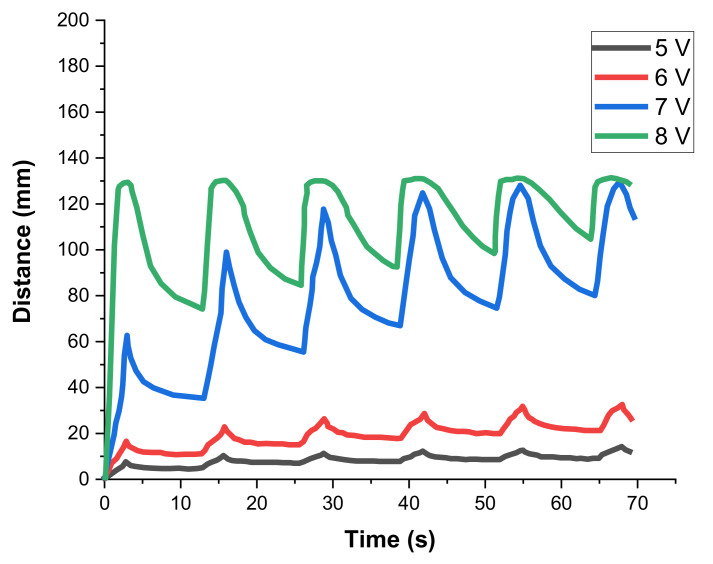
Bending distance of the adaptive structure at different voltages over the measurement duration.

Upon the application of different voltages, the bending distance of the composite structure varied. When the voltage was periodically switched off, the joule heating stopped and the SMA wire cooled down below the activation temperature, which led to the rebending of the composite, which was additionally supported by gravity (the influence of additional weights on re-deformation will be shown later) (see Figure 6a,b). Since cooling was constrained by the fact that the SMA was embedded in a thermally insulating LBR elastomer, the re-deformation process was delayed and showed a relaxation-like behavior. This behavior over the entire testing period is shown at different input voltages in Figure 7. The adaptive structure moved following the application of voltage followed by a relaxed return to its original position upon the removal of voltage, which was, however, constrained by the limited cooling of the SMA due to the thermal insulation of both the braided yarn shell and the elastomer matrix. The actuation cycles with 5 and 6 volts produced insufficient joule heating with only low bending deformation in the actuating curve, followed by a rather fast retreat to the original position in a relaxing curve. Higher bending deformations were attained at 7 and 8 volts. In comparison to the actuation at 5 V (peak distance of 7 to 14 mm), the actuation at 6 V showed a higher attainable bending distance (peak distance of 16 to 33 mm). Upon actuation at 7 V, it could be clearly observed that the first cycle of actuation (peak distance of 58 mm) had not already gained sufficient temperature increases to attain the SMA’s phase transition, as compared to the sixth cycle (peak distance 128 mm). From the third cycle on, the actuator attained the maximum deformation. Then, the actuated peaks henceforth reached a similar distance. However, the recoverable distance decreased in each subsequent cycle at 7 V and 8 V due to the thermal insulating effect mentioned above.

For the lower activation voltages, the SMA phase transition merely started, as the power input did lead to efficient heating of the SMA wire. At activation voltages of 7 and 8 V, the joule heating was much more efficient and, thus, the phase transition was fully completed within the fourth and fifth cycle, respectively. The maximum deformation distance of 130 mm corresponded to a deformation potential of 130% in this case. The time until the actuator reached its maximum deformation was about 1.8 s, whereas the relaxation of the sample required a much longer time and was not complete after 5 s.

The adaptive structure achieved the highest possible bending position among the voltages investigated at 8 V (peak distance 131 mm). The maximum bending distance for this voltage was already reached in the first cycle and remained at that same level in each subsequent cycle. Thus, the heat-induced martensite-to-austenite phase transformation of the SMA wire was able to be sufficiently fast, when activated at 8 V, during all five cycles.

Here, the adaptive structure retained the maximum deformed state for prolonged durations, but recovery to the original undeformed stage was more and more constrained in each subsequent cycle. Similar experiments with comparable findings were performed by other researchers but they made use of silicone rubber tubes as the “elastomer matrix” material [26].

In addition to the thermal insulation, which led to reduced cooling, another major reason for the fact that the adaptive structure did not retreat to the initial undeformed vertical position after SMA activation was considered to be the limited amount of elastic deformation energy stored within the very soft LBR-elastomer matrix. Thus, the elastomeric matrix could not generate sufficiently high support the re-deformation of the stiff SMA wire and to overcome the eventual frictional resistance generated by relative movement between the SMA wire surface and the hybrid glass-polypropylene yarn-braided shell.

The actuation would be faster if the experiment was performed in a pre-cooled condition of the specimen, and re-deformation might be stimulated by effective cooling; for example, with the use of a fan. However, if the soft actuator was used in a robotic system in order to lift a weight, for example, the presence of the mass at the lower end of the bending beam would additionally support re-deformation by gravity. This was subsequently studied.

**Figure 8:**
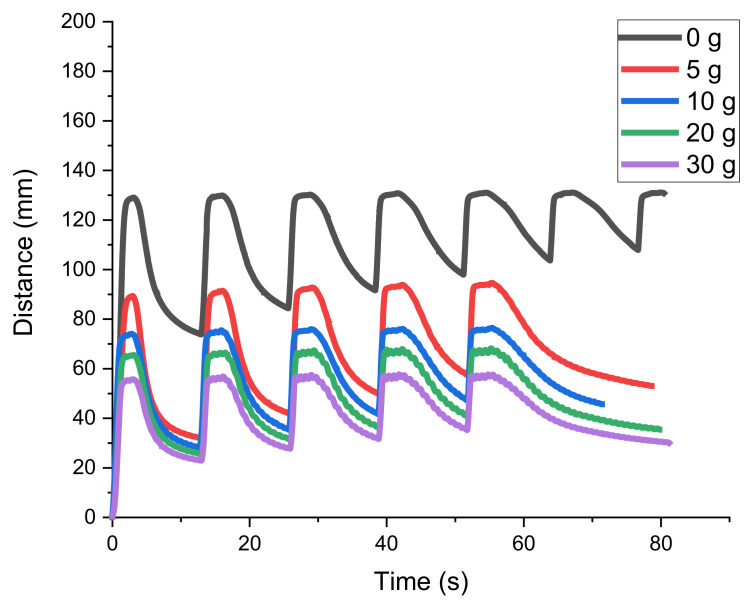
Actuation performance of the specimen with different weights on it at 8 V.

Weights of different mass were applied to the soft actuator in another set of trials to assess its suitability as a soft arm or gripper. The results are depicted in Figure 8.

Since the highest actuation (without load) was achieved by 8 V, this voltage was considered as a reference for the further experiments with the applied load. In every curve, with different loadings (5 g, 10 g, 20 g, and 30 g), it was observed that the relaxation peak reduced with the increasing of the applied weight (peak compared to no load curve at 8 V).

Moreover, the bending/de-bending cycles became much more uniform and re-deformation occurred faster. The applied weight acted as a pulley so the actuator beam returned to the original position rather than actual cooling of the SMA taking place. Comparable results were achieved by Kim et al., who used such composites to hold different objects and measured the performance of the SMA-integrated elastomeric actuator [27].

On the other hand, it could be observed that with the increasing of the weight, the maximum bending deformation achievable by actuation was also reduced. For a weight of 30 g, the motion amplitude was only one third of the unrestricted motion. This was, of course, a consequence of the very limited bending stiffness of the smart fiber-rubber composite, but the motion was still of significant amplitude. Thus, a weight of more than three times the actuator weight could be lifted, which is rather remarkable.

It was additionally observed that at weights of 10 g to 30 g, a small vibration, overlaying the basis movement, took place. This indicates that for larger blocking forces, an advanced control algorithm would be desirable.

### Physical Modelling and System Validation

**Figure 9:**
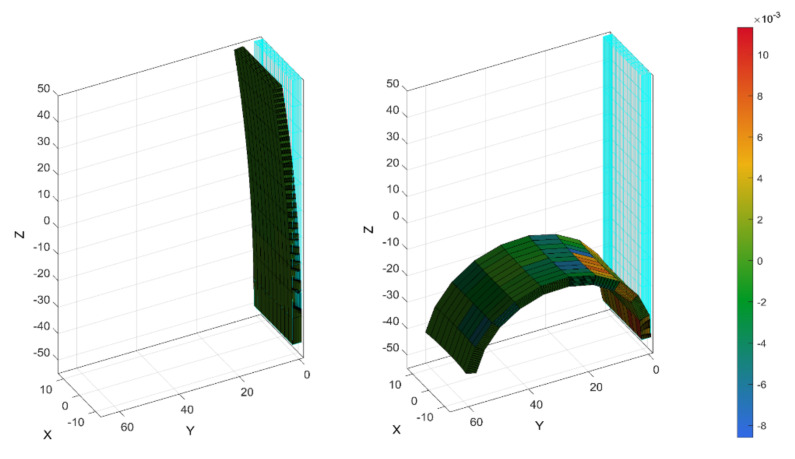
Simulation of bending actuation.

**Figure 10:**
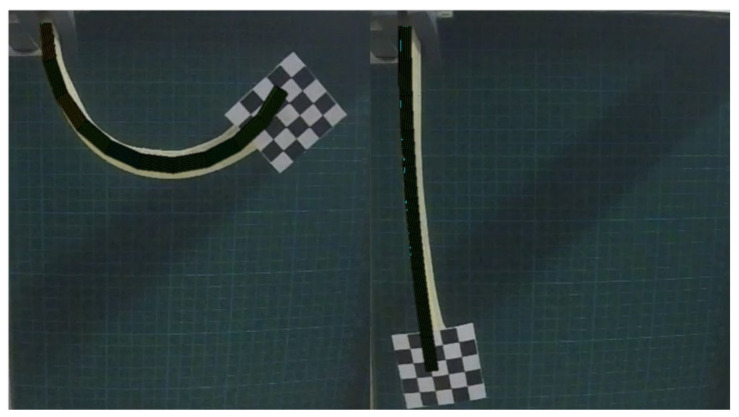
Comparison between the experimental observation and simulated bending deformation of the interactive fiber-elastomer composite.

As the presented physical system can be considered to mimic or substitute biological systems, we explored the use of modelling methodologies, such as those used for biological systems and tissue. The nonlinear finite element solver FEBio, controlled by the Gibbon toolbox, was used as a tool [28]. Therein, the soft actuator was modelled as a bending beam constituted of three layers: a passive elastomer matrix, a passive textile layer and an active textile layer (the active textile is the shape memory alloy hybrid yarn and the passive textile is the woven glass fiber), respectively. The geometrical dimensions were set as equal to the real dimensions. For the soft matrix material, the parameters of the hyperelastic Ogden material model (k = 175.5, c = 0.03475, m = 1.693) were fitted to match the experimental data from the tensile test in Figure 5a. The textile layer was modelled as solid mixture of a three-parameter Ogden material matrix with orthotropic fiber reinforcement. On top, the force exerted by the SMAs was implemented as a homogenized layer with the stress caused by the SMAs smoothed over the cross-section. The simulation results of an unconstraint bending deformation are shown in Figure 9. A comparison between the experimental actuation and the simulated actuation is provided in Figure 10, where optical images and simulated deformation images are overlapped.

The simulation results show good agreement with the experimental observations for the fully activated sample that was activated at 7 V. For higher SMA forces (achieved by higher actuation voltages), the simulation was not stable. Consequently, the use of the approaches developed within the biomechanics research community also shows promise for application in soft robotic systems. This is potential use is not restricted to the modelling of non-linear higher deformation problems; potential uses also exist in the development of adjustable experimental testing [29].

## 4. Conclusions

The main aim of this research work was to develop planar actuators based on SMA-integrated textile-reinforced liquid polybutadiene rubber and to evaluate its actuation performance by means of the joule heating process. Challenges were confronted to cure the liquid polybutadiene rubber matrix at a temperature that was lower than the phase transformation temperature of SMA. For this purpose, an LBR-formulation using ultra-fast accelerators had to be developed and an adapted liquid-mixing procedure had to be elaborated. To achieve this desired actuation, we used a shape memory alloy wire, which was braided by a hybrid yarn shell as a so-called “active textile” and integrated into the polypropylene glass fiber fabric (=”passive textile”) using the tailored fiber-placement technology. Then, the textile structure was compression molded with liquid butadiene rubber and subsequentially cured at low temperature to prevent SMA activation. The composites were characterized in terms of their electromechanical behavior as a function of the actuation voltage and applied weight. The maximum deformation (bending distance of 130 mm) was achieved at 8 V in the unloaded case, which was reduced to 90 mm, 70 mm, 65 mm and 57 mm with attached weights of 5 g, 10 g, 20 g and 30 g, respectively. It was found that the joule heating-based activation (=bending) occurred very fast, but the free rebending after actuation followed a relaxation-like process, which was mainly attributed to the constraint cooling of the SMA embedded in the sandwich structure. This inherent behavior is in line with findings of other authors using SMA in polymeric actuators. The actuation behavior of the unloaded composite structure was successfully simulated using the FEBio solver, where the elastomer matrix was represented by a simplified hyperelastic three-parameter Ogden model. With further improvements, the developed textile-integrated elastomeric composites might find application as soft robotic grippers, artificial muscles and even as soft prosthetics.

## Figures and Tables

**Table 1:** Properties of the liquid polybutadiene rubber (LBR 300) and shape memory alloy wire (SMA).

Property	Value and Unit
Liquid Butadiene Rubber (LBR)	
Molecular weight	45,000 g/mol
Viscosity	280 Pa-s at 38 °C
Glass transition temperature (T_g_)	−95 °C
Shape Memory Alloy (SMA)	
Young’s modulus	austenite approx. 83 GPa martensite approx. 28 to 41 GPa
Yield strength	austenite 195 to 690 MPa martensite 70 to 140 MPa
Ultimate tensile strength	fully annealed 895 MPa work-hardened 1900 MPa
Elongation at failure	fully annealed 25 to 50% work-hardened 5 to 10%

**Table 2:** LBR rubber formulation curable at 75 °C.

Compound	Quantity (Parts per Hundred Rubber (phr))
Liquid butadiene rubber (LBR-300)	100
Zinc oxide	3
Stearic acid	2
CBS	1.5
Sulfur	2.2
DPG	1
Rhenocure	2

## Data Availability

Not applicable.
